# Characterizing circular peptides in mixtures: sequence fragment assembly of cyclotides from a violet plant by MALDI-TOF/TOF mass spectrometry

**DOI:** 10.1007/s00726-012-1376-x

**Published:** 2012-08-14

**Authors:** Hossein Hashempour, Johannes Koehbach, Norelle L. Daly, Alireza Ghassempour, Christian W. Gruber

**Affiliations:** 1 Center for Physiology and Pharmacology, Medical University of Vienna, Schwarzspanierstrasse 17, 1090 Vienna, Austria; 2 Medicinal Plants and Drugs Research Institute, Shahid Beheshti University, G.C. Evin, Tehran, Iran; 3 School of Pharmacy and Molecular Sciences, Centre for Biodiscovery and Molecular Development of Therapeutics, Queensland Tropical Health Alliance, James Cook University, Cairns, 4878 Australia

**Keywords:** *Viola ignobilis*, Circular, Cystine-knot, Oxidative folding, Vigno, Peptidomics

## Abstract

Cyclotides are a very abundant class of plant peptides that display significant sequence variability around a conserved cystine-knot motif and a head-to-tail cyclized backbone conferring them with remarkable stability. Their intrinsic bioactivities combined with tools of peptide engineering make cyclotides an interesting template for the design of novel agrochemicals and pharmaceuticals. However, laborious isolation and purification prior to de novo sequencing limits their discovery and hence their use as scaffolds for peptide-based drug development. Here we extend the knowledge about their sequence diversity by analysing the cyclotide content of a violet species native to Western Asia and the Caucasus region. Using an experimental approach, which was named *sequence fragment assembly* by MALDI-TOF/TOF, it was possible to characterize 13 cyclotides from *Viola ignobilis*, whereof ten (vigno 1–10) display previously unknown sequences. Amino acid sequencing of various enzymatic digests of cyclotides allowed the accurate assembly and alignment of smaller fragments to elucidate their primary structure, even when analysing mixtures containing multiple peptides. As a model to further dissect the combinatorial nature of the cyclotide scaffold, we employed in vitro oxidative refolding of representative vigno cyclotides and confirmed the high dependency of folding yield on the inter-cysteine loop sequences. Overall this work highlights the immense structural diversity and plasticity of the unique cyclotide framework. The presented approach for the sequence analysis of peptide mixtures facilitates and accelerates the discovery of novel plant cyclotides.

**Electronic supplementary material:**

The online version of this article (doi:10.1007/s00726-012-1376-x) contains supplementary material, which is available to authorized users.

## Introduction

Cyclotides are a unique class of cysteine-rich macrocyclic mini-proteins of about 30 amino acids in size that are defined by a head-to-tail cyclized backbone and three disulfide bonds in a knotted arrangement referred to as cyclic cystine-knot (CCK) motif (Craik et al. 1999). Their knotted structure makes them exceptionally stable against thermal, chemical and enzymatic degradation (Colgrave and Craik 2004). Cyclotides have been discovered and isolated from plants of the violet (Violaceae), coffee (Rubiaceae), cucurbit (Cucurbitaceae) and legume family (Fabaceae) (Poth et al. 2010). Their distribution within the plant kingdom still remains unclear (Gruber 2010), but they are expected to be far more widespread and the number of different cyclotides may be around 50,000 (Gruber et al. 2008; Simonsen et al. 2005) making them one of the largest peptide classes within plants. In agreement with their anticipated number, recent studies report the presence of more than 70 different cyclotides within one single species (Seydel et al. 2007; Gründemann et al. 2012). The first cyclotide kalata B1 was discovered from “kalata-kalata”, a decoction from leaves of *Oldenlandia affinis*, which has been used as a remedy during childbirth in African ethnomedicine due to its uterotonic activity (Gran 1970; Gruber and O’Brien 2011). In line with their reported antibacterial (Tam et al. 1999), antifouling (Göransson et al. 2004), anthelmintic (Colgrave et al. 2008) and insecticidal properties (Jennings et al. 2001; Gruber et al. 2007a; Barbeta et al. 2008) their native function seems to be part of the plant defence system.

**Fig. 1:**
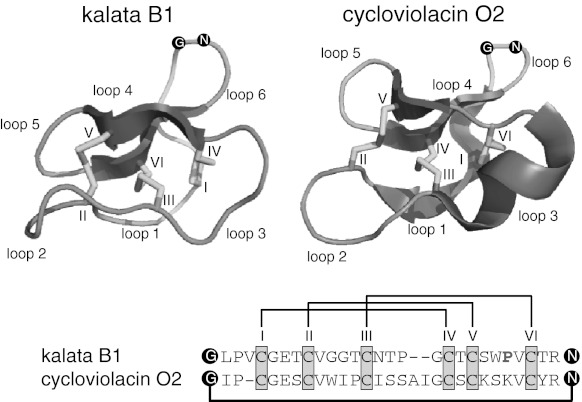
Ribbon structures of the cyclotides kalata B1 (*left panel*), a representative of the Möbius subfamily and cycloviolacin O2 (*right panel*) belonging to the bracelet subfamily are shown as *cartoons*. The unique cyclic cystine-knot (CCK) motif with three conserved disulfide bonds (*yellow*) and the cyclized backbone (*black dots* and *connecting line*) as well as typical secondary structure elements of α-helices (*blue*) and β-sheets (*red*) and their respective sequences are shown (PDB code: 1NB1 and 2KNM, respectively). The disulfide connectivity C_I–IV_, C_II–V_ and C_III–VI_ has been indicated with *black lines* (color figure online)

As a key feature, cyclotides are amenable to various amino acid changes by peptide engineering, which highlights the flexibility and plasticity of the cyclotide framework (Clark et al. 2006). Thus, their high sequence diversity is extensively under investigation for being utilized as scaffolds in the development of agrochemicals and pharmaceuticals (Henriques and Craik 2010). Besides these distinct differences in the sequences of the so-called inter-cysteine loops, cyclotides can be divided into two subfamilies, i.e., Möbius or bracelet type cyclotides based on the presence or absence of a *cis*-Pro residue in loop 5 (Fig. 1) (Craik et al. 1999). These differences have further implications regarding their physico-chemical properties. Whereas most Möbius cyclotides are slightly negatively charged or have an overall net-charge of zero, bracelet cyclotides are usually multiply positively charged. This ultimately influences their chemical behaviour and amenability to sequencing and oxidative folding, which are still challenges, in particular for bracelet cyclotides.

Usually, amino acid sequencing of cyclotides is performed after enzymatic digestion of peptides that have been laboriously purified by reversed-phase high performance liquid chromatography (RP-HPLC) to produce single linearized peptides that are amenable to tandem mass spectrometry (MS) analysis. However, the complexity of cyclotide plant extracts, which comprise dozens of distinct peptides, limits their analysis and characterization by standard MS analysis. Using endoproteinase GluC (endo-GluC), cyclotides are mostly cleaved to yield a single (‘ring-opened’) peptide fragment due to a conserved glutamic acid in loop 1, whereas the use of trypsin and chymotrypsin usually yields several fragments due to multiple cleavage sites. When applied to the analysis of cyclotide mixtures as they occur in plant extracts, mass spectra may be confusing and hard to evaluate caused by fragment ion overlays. Hence the application of combinations of digests to obtain peptide-specific fragments and the subsequent accurate assembly of sequence fragments may overcome this issue. Particularly for bracelet cyclotides this is of importance since until now the majority (~70 %) of more than 200 published cyclotide sequences accessible on CyBase (Wang et al. 2008b) belong to this subfamily.

Besides the complexity of cyclotide sequence analysis, another issue associated with their great diversity is their chemical and biological synthesis. Previous studies have shown that different enzymes seem to be involved in backbone cyclization and disulfide bond formation (Gruber et al. 2007b; Saska et al. 2007) during biosynthesis of these gene-encoded peptides *in planta*. However, the community still lacks clarity about this process, in particular with respect to the *sequence*-*folding relationship*, i.e., how the inter-cysteine sequences of different cyclotides can influence the formation of the native CCK-motif and hence determine their folding yield. As a consequence, in vitro oxidative folding is still a major challenge in cyclotide engineering. Whereas high-yield chemical synthesis and folding of Möbius cyclotides is possible (Daly et al. 1999), obtaining correctly folded bracelet cyclotides is much more difficult and yields of about 10–40 % or less of native peptide are common (Leta Aboye et al. 2008; Wong et al. 2011). However, chemical synthesis of cyclotides is an important tool to obtain sufficient peptide material for bioactivity studies.

Our aim is to characterize plant cyclotides from *Viola ignobilis*, a native Iranian species of the violet family that was recently discovered to contain cyclotides (Hashempour et al. 2011). As a rich source for representatives of both cyclotide subfamilies, we analysed cyclotide-containing fractions using matrix-assisted laser desorption ionization-time of flight (MALDI-TOF) MS and tandem MS analytics. Together with the use of different enzymatic digests and detailed analysis of mass spectra, full peptide sequence coverage could be achieved by assembling and aligning various sequence fragments. This approach, which we called *sequence fragment assembly* turned out to be a powerful tool for cyclotide identification and de novo sequencing even when analysing mixtures. To dissect the influence of cyclotide sequence variability with respect to the formation of their native structure, we performed oxidative refolding experiments using representative vigno (*V. ignobilis*) cyclotides comprising distinct, but subtle differences in their inter-cysteine loop sequences. Overall the characterization of these novel cyclotides highlights their enormous sequence variability and the proposed sequencing methodology may overcome limitations in the discovery of novel representatives of this unique class of circular plant peptides.

## Materials and methods

### Plant collection, extraction and RP-HPLC fractionation

Aerial parts of *V. ignobilis* Rupr. were collected in the mountains at an altitude of 1,500–2,500 m around the village of Negarestan in the region of East Azerbaijan (Iran) in spring 2010. A voucher specimen was identified and deposited at the Institute of Medicinal Plants and Drug Research, Iran (MPH-1917). The dried plant material (~500 g) was ground prior to solvent extraction with a mixture of MeOH:CH_2_Cl_2_ (1:1; v/v) overnight under continuous agitation at 20 °C. After adding of 0.5 volume water the aqueous phase was concentrated on a rotary evaporator prior to freeze drying, yielding what is further referred to as crude extract. The crude extract was dissolved in 0.1 M NH_4_HCO_3_ buffer (pH ~ 8.1) and immediately used for solid-phase extraction (SPE). C_18_ SPE cartridges (Macherey-Nagel, Chromabond; 10 g; 50 mL) were activated with 1 bed volume of MeOH and subsequently equilibrated with 1 bed volume of aqueous 1 % FA. After application of the extract, the cartridges were washed with 1 bed volume of 1 % FA. Putative cyclotide containing fractions of 50 and 80 % EtOH were collected and freeze dried. After dissolving in 1 % FA they were fractionated using preparative and semi-preparative RP-C_18_ HPLC (Knauer, Eurospher I 5 μm; 250 × 16.1 mm; 100 Å) using a Knauer 1200 series unit, with an isocratic flow of 30 % acetonitrile: H_2_O (v/v) at a flow rate of 8 mL min^−1^. Fractions were collected manually by UV detection at 210 nm. All samples were extracted by avoiding prolonged exposure to high pH and sample heating to reduce the risk of Asn deamidation.

### Reduction, alkylation and enzymatic digest

Prior to MS analysis, cyclotides were enzymatically digested to produce linearized fragments following reduction and alkylation of Cys-residues. Lyophilized samples (~0.5 μg peptide) were dissolved in 0.1 M NH_4_HCO_3_ buffer (pH 8.2) and 20 μL aliquots were reduced by adding 2 μL of 10 mM dithiothreitol and were incubated at 20 °C for 30 min. Alkylation was carried out by adding 4 μL of 100 mM iodoacetamide to the reduced samples and incubating for 10 min at 20 °C. After a second incubation step for 10 min with 1 μL of 10 mM dithiothreitol to quench the reaction with iodoacetamide, 2 μL of trypsin, endo-GluC and/or chymotrypsin (all Sigma-Aldrich, Austria) at concentrations of 0.1–0.5 μg μL^−1^ were added. All digests were incubated at 37 °C between 3 and 16 h, quenched with concentrated acetic acid (final concentration 3 %) and stored at 4 °C−20 °C until further analysis.

### MALDI-TOF/TOF analysis and peptide sequencing

Analysis of crude, reduced/alkylated and digested samples were performed on a MALDI-TOF/TOF 4800 Analyser (AB Sciex, Canada) operated in reflector positive ion mode acquiring 2,000–3,600 total shots per spectrum with a laser intensity set between 3,200 and 3,800. MS and MS/MS experiments were carried out using α-cyano-hydroxyl-cinnamic acid matrix at a concentration of 5 mg mL^−1^ in 50 % (v/v) acetonitrile. 0.5 μL of each sample was mixed with 3 μL of matrix solution and the mixture was spotted onto the target plate. Tandem mass spectra were acquired using laser energy of 1 kV with and without the use of collision-induced dissociation and processed using the Data Explorer Software. Cyclotides were identified by sequence fragment assembly (as explained below) and manual peptide sequencing. Automated database searches using the ERA-tool (Colgrave et al. 2010) and DeNovoExplorer software were used to compare manual annotated sequences. The MS/MS spectra were examined and sequenced based on assignment of the N-terminal b-ion and C-terminal y-ion series. The disulfide connectivity of C_I–IV_, C_II–V_ and C_III–VI_ was assigned based on homology with published sequences.

### Oxidative refolding of cyclotides

Cyclotides were purified by RP-HPLC on a Dionex Ultimate 3000 HPLC unit (Dionex, Netherlands) using semi-preparative (250 × 10 mm) and analytical (250 × 4.6 mm) Kromasil C_18_ columns (5 μm; 100 Å) with linear gradients of 0.1–1 % min^−1^ or isocratic flow of 25–35 % buffer B (90 % acetonitrile in ddH_2_O, 0.08 % TFA) at flow rates of 3 and 1 ml min^−1^, respectively. The control peptide kalata B1 was isolated from *Oldenlandia affinis* extract as described earlier (Gründemann et al. 2012). The same procedure was applied for the purification of cycloviolacin O2 from *Viola odorata*. Reduction was performed as described above and stopped after 30 min incubation by adding concentrated TFA (Sigma-Aldrich, Austria) and samples were immediately subjected to HPLC purification. Folding of 60 μL aliquots, containing 2.5–10 μM peptide, was performed at final concentration of 2 mM reduced (GSH) and 0.1 mM oxidized (GSSG) glutathione (Sigma-Aldrich, Austria). Freeze-dried aliquots were resolved in three different folding-buffers, i.e., 25 and 75 % isopropanol (Roth, Germany) and 35 % DMSO/5 % dodecyl-β-maltoside (DBM) in 0.1 M NH_4_HCO_3_ buffer (pH 8.2). For control experiments with cycloviolacin O2 the folding conditions included final concentrations of 2 mM GSH and 2 mM cystamine in 35 % DMSO/5 % DBM buffer and GSH/cystamine (2/2 mM) in 0.1 M Tris–HCl buffer (pH 8.5) at 4 °C and 20 °C. Aliquots were analysed at several time points (15 min, 1 h and 24 h) after incubation at 20 °C. Folding reaction was quenched by adding 1 μL of concentrated TFA and samples were analysed by RP-HPLC on an Aeris Peptide XB-C_18_ (150 × 2.1 mm; 3.6 μm; 100 Å) column (Phenomenex, Germany) at a flow rate of 0.3 ml min^−1^ with a gradient of 2 % min^−1^ buffer B. Folding yields were determined using the peak integration tool of Chromeleon software 6.8 with a peak detection limit set at 0.07 × signal (mAU at 214 nm) × retention time (min). Folding kinetic graphs and calculations of rate constant and half-time were prepared using the one-phase association fit in GraphPad Prism 5 software.

### Cyclotide homology modelling

The structural models of vigno 1, 2 and 10 were modelled using the CycloMod application for cyclotide structure modelling within Cybase (http://www.cybase.org.au/). The models were generated using Modeller 9.10 and analysed by Molprobity (Davis et al. 2007). The percentage of residues in the most favoured Ramachandran region and the Molprobity scores are: vigno 1 (92.59 % and 2.71), vigno 2 (89.29 % and 2.75) and vigno 10 (89.66 % and 3.27).

## Results and discussion

The discovery and hence the pharmaceutical value of cyclotides is limited by an efficient and reliable protocol for peptide sequence analysis, in particular in crude plant extracts and fractions that contain mixtures of different cyclotides. Therefore, the main goal of this study was to describe a robust method for cyclotide sequence characterization using MALDI-TOF/TOF analytics.

### Identification of novel cyclotides from *Viola ignobilis*

**Fig. 2:**
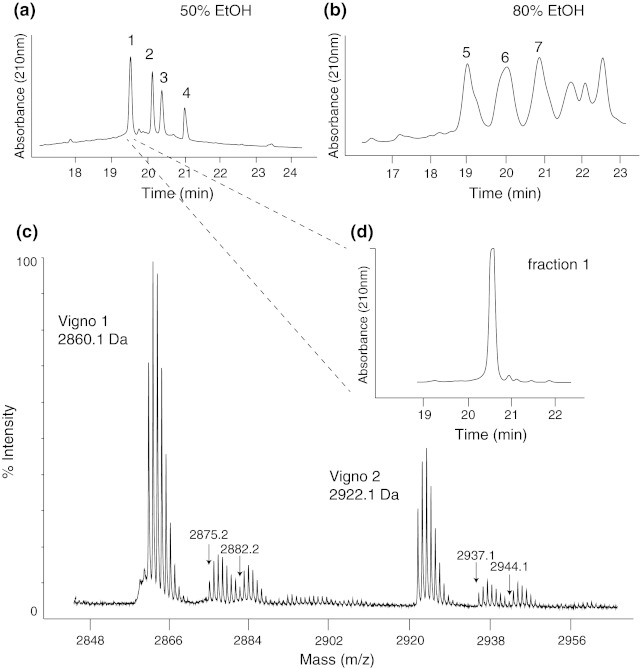
HPLC fractionation of cyclotides from *Viola ignobilis* extract. Analytical HPLC traces of the 50 % (**a**) and 80 % (**b**) ethanolic solid-phase extracts yielding seven cyclotide-containing subfractions (labelled *1*–*7*). (**c**) MALDI-TOF/TOF spectrum of fraction 1 indicating the masses of several cyclotides including the most abundant Möbius cyclotides vigno 1 and vigno 2, which are co-eluting (**d**) on analytical HPLC

*Viola ignobilis*, a violet plant that frequently occurs in mountainous regions of Iran has recently been described to contain cyclotides (Hashempour et al. 2011) and this is consistent with previous studies showing that all Violaceae plants analysed to date express this unique class of cyclic mini-proteins. According to established extraction protocols it was possible to identify and isolate several cyclotide-containing fractions from *V. ignobilis*. Figure 2 shows the seven fractions obtained by RP-HPLC fractionation of the initial two solid-phase extracts. Exemplarily, a MALDI-TOF spectrum of one fraction (Fig. 2c) indicates the presence of multiple cyclotides whereas standard HPLC analysis is not powerful enough to resolve those co-eluting cyclotides (Fig. 2d). This is a common scenario and usually requires further purification prior to MS sequencing. However in the case of low sample amounts, laborious purification may lead to sample loss and the use of mixtures might be inevitable. Hence the aim was to develop a protocol to perform MALDI-TOF/TOF-based sequence characterization using semi-pure fractions containing at least two cyclotides. Subsequently it was possible to identify 13 cyclotides (vigno 1–10, varv A, cycloviolacin O2 and cO9) in seven HPLC fractions (Fig. 2a, b).

### De novo cyclotide sequencing using ‘sequence fragment assembly’

Cyclotide-containing mixtures were chemically modified to yield S-carbamidomethylated Cys-residues and digested to produce linear peptides amenable to fragmentation by tandem MS. Completely reduced and alkylated samples were digested using single enzymes or combinations of trypsin, endo-GluC and chymotrypsin. Resulting mass spectra were manually analysed by assigning N-terminal b- and C-terminal y-ions. Most novel cyclotide sequences were independently confirmed by automatic database searches using the ERA-tool (Colgrave et al. 2010) and DeNovoExplorer software.

**Fig. 3:**
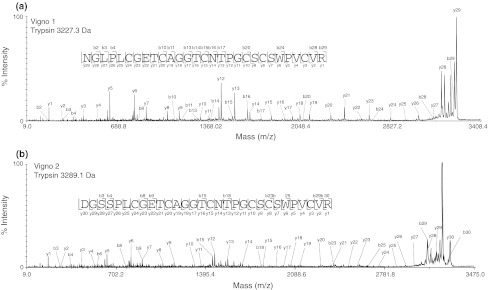
MALDI-TOF/TOF sequencing of co-eluting vigno 1 and 2. MS/MS spectra of the precursor masses of 3,227.3 Da (**a**) and 3,289.1 Da (**b**) of a tryptic digest (reduced and S-carbamidomethylated) of fraction 1 (see Fig. 2) is shown. Observed C-terminal y- and N-terminal b-ions that allowed sequence characterization are labelled

**Fig. 4:**
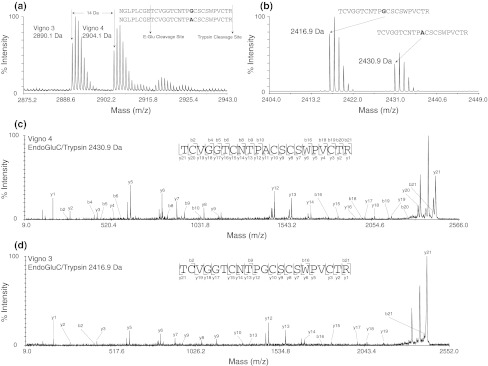
MALDI-TOF/TOF identification of the co-eluting peptides vigno 3 and vigno 4. The difference of 14 Da can be observed in crude (**a**) and within the combined trypsin and endoproteinase GluC digest (**b**) of fraction 4. MS/MS sequencing of the endo-GluC/trypsin-digested precursors with 2430.9 Da (**c**) and 2416.9 Da (**d**), respectively, allowed unambiguous assignment of the sequences of these two peptides

Cyclotide sequences are generally obtained from pure peptides by enzymatic digestion and interpretation of tandem mass spectra. For co-eluting Möbius cyclotides in mixtures, such as vigno 1 and vigno 2 (Fig. 2), it was possible to obtain the full, unambiguous sequences by evaluating the ion-fragmentation pattern and by alignment of two independent digests using trypsin or endo-GluC (Fig. 3; Supplementary Table S1). In cases where single digests yielded incomplete fragment ion coverage, a combination of trypsin and endo-GluC digests was applied to generate smaller fragments and their analysis enabled complete sequence interpretation as shown for vigno 3 and 4 (Fig. 4, Supplementary Fig. S1; Supplementary Table S1). This procedure of reduction, alkylation and enzymatic digest provided also good sequence coverage of other Möbius cyclotides in mixtures namely varv A and vigno 5 (Supplementary Fig. S2; Supplementary Table S1).

**Fig. 5:**
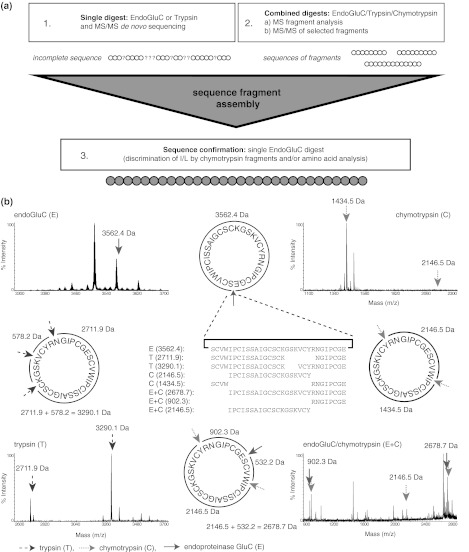
Sequence fragment assembly approach for vigno 6. **a** An overview of the sequence fragment assembly workflow that has been used to elucidate cyclotide sequences in mixtures is presented. **b** The combination of single trypsin, chymotrypsin and endoproteinase GluC and a combination of chymotrypsin/endo-GluC digests of fractions containing multiple cyclotides together with the alignment of partial sequences and assembling of peptide-specific fragments allowed the discrimination and unambiguous assignment and elucidation of the cyclotide sequences. MS spectra of four digests using endo-GluC (*upper left panel*), chymotrypsin (*upper right*), trypsin (*lower left*) and a combination of endoproteinase GluC and chymotrypsin (*lower right*) are shown for vigno 6. The cleavage sites and resulting peptide fragments of the different enzymes are indicated by *arrows* (trypsin: *blue/dashed line*, chymotrypsin: *green/dotted line*, endo-GluC: *red/straight line*). The alignment of obtained MS sequence fragments (*middle*) together with MS/MS sequence data of selected precursors (see Fig. 6) allows the unambiguous sequence elucidation of the novel cyclotide vigno 6 (color figure online)

**Fig. 6:**
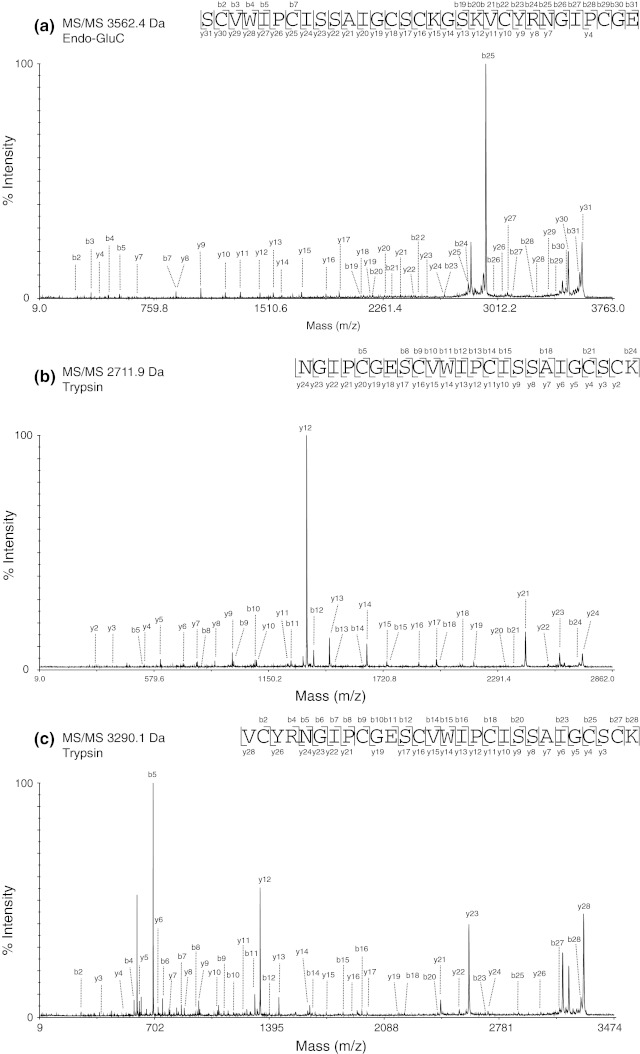
MS/MS sequencing of vigno 6. Three MS/MS spectra of (**a,** **b**) the precursors with the molecular weight of 2,711.9 and 3,290.1 Da, respectively, from a tryptic digest and (**c**) the linearized cyclotide precursor with a molecular weight of 3,265.4 Da from an endoproteinase GluC digest are shown. The sequences were obtained by assigning the y- and b-ions series

By contrast, this methodology did not yield evaluable mass spectra for bracelet cyclotides. A linearized bracelet cyclotide using endo-GluC very often results in incomplete fragmentation making it impossible to assign. Tryptic digests of fractions containing multiple bracelet cyclotides on the other hand resulted in unclear fragmentation patterns due to multiple enzyme cleavage sites and hence fragment-ion overlay. Since cyclotides of the bracelet subfamily often contain multiple basic residues (Arg and Lys), a tryptic digest typically yields fragments corresponding to single, double or multiple cleaved peptides (Supplementary Table S2). Due to the high sequence homology of cyclotides, the same fragment sequence may appear in more than one peptide and, therefore, in cyclotide mixtures these fragments have the same mass. To circumvent this problem, single and double digests combining chymotrypsin and endo-GluC were performed. This resulted in peptide fragments of distinct molecular weight. By combining the sequence information from the tryptic fragments and alignment of the distinct fragments it was possible to assemble the full cyclotide sequence (Figs. 5, 6; Supplementary Fig. S3, Supplementary Fig. S4). Finally each sequence had to be confirmed by assignment of the linearized endo-GluC digested cyclotides. Exemplarily, a flowchart of the sequence fragment assembly approach and the sequence elucidation of the bracelet cyclotide vigno 6 have been presented (Figs. 5, 6). The tryptic digest of fraction 5 or 6 (Fig. 2b) leads to the two fragments of 2,711.9 and 3,290.1 Da that may originate from several cyclotides, e.g., vigno 6, vigno 8 or cycloviolacin O2 (Supplementary Table S2). On the other hand, the chymotrypsin digest and a combination of chymotrypsin and endo-GluC yields fragments with distinct molecular weights and by combining the sequence information from the tryptic peptides with alignment of the chymotrypsin/endo-GluC fragments it was possible to assemble the full sequence.

Table 1 shows all cyclotide sequences from *V. ignobilis* that were elucidated by the sequence fragment assembly approach. The combination of several single and double digests of fractions containing multiple cyclotides and the alignment of partial sequences and assembling of peptide-specific fragments allowed the discrimination and unambiguous assignment and elucidation of the distinct cyclotide sequences. This approach ultimately led to the identification of 13 novel cyclotides, whereof ten display previously unknown sequences (Table 1). The use of multiple enzymes and varying combinations thereof together with MALDI-TOF/TOF analysis will overcome major limitations of cyclotide de novo sequencing and facilitate the discovery of novel sequences within peptide mixtures. MS/MS spectra and summaries of all digested fragments used for the sequence determination of all cyclotides from *V. ignobilis* are available as Supplementary Data (Supplementary Fig. S1, Supplementary Fig. S2, Supplementary Fig. S3, Supplementary Fig. S4, Supplementary Table S1, Supplementary Table S2). To identify cyclotides in mixtures using MALDI-TOF/TOF analysis is a powerful tool for an efficient sequence elucidation and the discovery of novel cyclotide sequences. The identification of ten novel cyclotides from *V. ignobilis* supports the evidence that cyclotides are one of the largest peptide classes within plants with immense sequence diversity in their inter-cysteine loops built around the stable CCK frame (Supplementary Fig. S5).

**Table 1:**
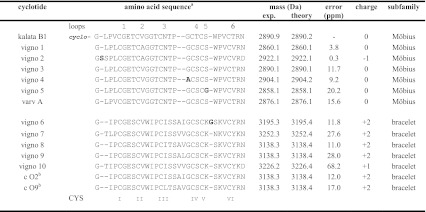
Sequence alignment of cyclotides identified from *Viola ignobilis*

^a^Isobaric amino acids Leu/Ile were assigned based on chymotrypsin digests (see Supplementary Figures S9 and S10) or based on homology to published sequences; novel sequence motifs are highlighted in bold

^b^Cycloviolacin

### Sequence variation of novel vigno cyclotides

The 13 identified peptides from *Viola ignobilis* belong to both subfamilies (Table 1). One Möbius cyclotide, known as varv peptide A, has been previously isolated from *Viola arvensis* (Claeson et al. 1998), as well as two bracelet cyclotides, known as cycloviolacin O2 and O9, have been originally found in *Viola odorata* (Craik et al. 1999). This is not surprising since some cyclotides such as varv peptide E (=cycloviolacin O12), occur in many different *Viola* species such as *V. tricolor, V. odorata*, *V. arvensis, V. bashoanensis*, *V. yedoensis* and *V. abyssinica* and, therefore, seem to be genus-specific. Besides these rather rare examples of inter-genus identity, each single plant species seems to express an abundant array of specific cyclotides. In *V. ignobilis* the most abundant Möbius cyclotides are vigno 1 and vigno 2. Sequence analysis of these two peptides revealed the presence of an AGGT motif in loop 2 which was recently also described for *V. abyssinica* cyclotides (Yeshak et al. 2011).

Besides the conserved six Cys-residues and the glutamic acid (E) in loop 1, all six Möbius cyclotides have the same typical GET motif in loop 1 and a serine in loop 4, which is conserved within all newly identified cyclotides from *V. ignobilis*. Furthermore, the GES motif in loop 1 and the VWIP motif in loop 2 are conserved within all bracelet cyclotides from *V. ignobilis*. The differences and novelties are within loop 3, 5 and 6 which are known to show the highest amino-acid variability (Supplementary Fig. S5; Table 1). For vigno 2 a novel sequence motif for loop 6 of cyclotides, VRDGSSPL, has been discovered. Although all amino acids are known to occur in this loop, the presence of two serine residues next to each other has not been reported hitherto. The presence of two serine residues and an aspartic acid makes this loop more hydrophilic and confers the peptide with an overall net charge of −1. To distinguish between Asn and Asp residues in loop 6 of vigno 2 and vigno 10, we have additionally analysed the molecular weight and isotopic distribution of diagnostic fragment ions (Poth et al. 2010) (Supplementary Figure S6). Further sequencing of the co-eluting Möbius cyclotides vigno 3 and vigno 4 (Fig. 2) revealed the presence of an alanine or glycine within loop 3 and corresponds to the mass difference of 14 Da in the crude sample (Fig. 4a). A combined digest using trypsin and endo-GluC yields the fragments of 2,416.9 and 2,430.9 Da that allowed the sequence determination and confirming the difference of a glycine (NTPG) and alanine (NTPA) at the last position of loop 3. This is to our knowledge the first report of an alanine residue at this position and expands the known possibilities at this position which was primarily thought to be a conserved glycine (Craik et al. 1999). Within the sequence of vigno 5 a glycine at the first position of loop 5 was found, which so far has only been shown for bracelet cyclotides such as cycloviolacin Y1-3 (Wang et al. 2008a) and tricyclon A and B (Mulvenna et al. 2005). Vigno 6 shows the very common KSKV sequence for loop 5, which is intersected by a glycine, KGSKV. The identification of ten novel peptides underlines the high flexibility and sequence variability caused by single amino acid changes at various positions. It is obvious that sequence variability accounts for different biological and chemical behaviour due to varying physico-chemical properties. As an example, we decided to characterize the in vitro oxidative refolding properties of representative vigno cyclotides, since it is a valuable model to analyse their sequence-folding relationship, which has broader implications on the synthesis and design of cyclotides as tools in pharmaceutical applications.

### Sequence-folding relationship of novel vigno cyclotides

The influence of certain residues on oxidative folding and the correct formation of the native disulfide bonds of cyclotides are still not fully understood. In the current study it has been our particular interest to elucidate the sequence-folding relationships of three vigno cyclotides with respect to their yield and folding kinetics using different folding conditions. Therefore, the oxidative refolding of the most abundant Möbius cyclotides in *V. ignobilis*, vigno 1 and vigno 2 was studied, in comparison to the prototypic cyclotide kalata B1, and the bracelet cyclotide vigno 10 in comparison to cycloviolacin O2, a well-studied bracelet cyclotide isolated from *V. odorata*.

**Fig. 7:**
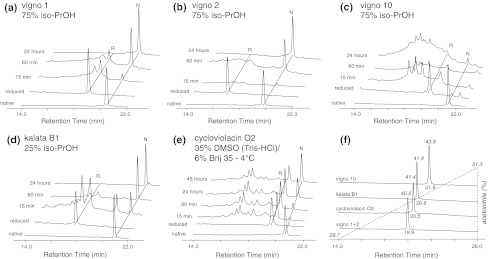
Refolding of cyclotides. RP-HPLC traces of native, reduced and refolded peptides under conditions leading to the highest yields of vigno 1 (**a**), vigno 2 (**b**), vigno 10 (**c**), kalata B1 (**d**) and cycloviolacin O2 (**e**) are offset aligned for clarity. The folding of vigno peptides and kalata B1 (**a**–**d**) was performed in 0.1 M NH_4_HCO_3_ at 20 °C and cycloviolacin O2 (**e**) folding was carried out in Tris-buffer at 4 °C (see “Materials and methods” for further details). **f** Difference in hydrophobicity of all peptides used in this study is shown

To provide material for the folding studies all three vigno cyclotides and the control cyclotides kalata B1 and cycloviolacin O2 were purified and reduced. All native and reduced peptides were analysed by MALDI-MS and RP-HPLC confirming their high purity (>95 %) and the cysteine oxidation state (Fig. 7, Supplementary Fig. S7, Supplementary Table S3). As expected the reduced cyclotides showed a mass shift of 6 Da on MALDI-MS, indicating the complete reduction of the three native disulfide bonds. This results in decreased overall hydrophobicity and hence the peptides eluted significantly earlier from the reversed-phase column (Fig. 7f).

Based on previous studies on kalata B1 (Daly et al. 1999) buffers containing 0.1 M ammonium-bicarbonate buffer with low (25 %) and high (75 %) concentration of isopropanol were used in the initial folding studies. High concentrations of alcohol were recently shown to favour folding of a bracelet cyclotide (Wong et al. 2011). In addition, a buffer containing 35 % DMSO and 5 % dodecyl-β-maltoside (DBM) was used; it has been shown for cycloviolacin O2 that a buffer containing DMSO and a non-ionic detergent yields reasonable amounts of folded peptide (Leta Aboye et al. 2008). All buffers contained the disulfide shuffling redox agents reduced (GSH) and oxidized (GSSG) glutathione. After dissolving the reduced peptides, the refolding process was monitored by taking aliquots at several time-points (15 min, 1 and 24 h). After 24 h incubation at 20 °C, refolding was stopped by quenching the reaction with concentrated TFA and samples were subjected to RP-HPLC (Fig. 7; Supplementary Fig. S7) and MALDI-MS analysis (Supplementary Table S3). Generally refolding starts immediately after the reduced peptides have been dissolved in the folding buffer, which can be determined by disappearance of the peak in the HPLC chromatograms corresponding to the reduced cyclotides. Refolding data were analysed by measuring the area under curve of the peaks corresponding to the reduced and native peptides and plotting the folding yields in percentage versus time of incubation (Fig. 7, Supplementary Fig. S7; Table 2). In particular for the Möbius cyclotides one can observe an increase of the peak corresponding to the native cyclotide after 15 min and the final yield was measured after 24 h of folding. As listed in Table 2, folding of the Möbius cyclotides vigno 1, vigno 2 and kalata B1 led to respectable yields between ~30 and 90 % of refolded peptides, favoured by higher isopropanol concentrations versus the DMSO/detergent buffer. This is consistent with previous studies and the fact that the hydrophobic solvent appears to stabilize the surface exposed hydrophobic patches of cyclotides (Daly et al. 1999). To compare the folding kinetics of each cyclotide the rate constant (*k*_*n*_) and the half-time (*t*_1/2_) of appearance of the native species were calculated. For the three Möbius cyclotides the half-time of appearance of the native peak was between 25 and 96 min (Table 2). Folding of bracelet cyclotides appears to be more complex and difficult under in vitro conditions as has previously been reported (Aboye et al. 2011; Gunasekera et al. 2009). Accordingly, the overall refolding yield of the bracelet cyclotides vigno 10 and cycloviolacin O2 in the two isopropanol buffers or the standard DMSO/detergent buffer (=35 % DMSO/5 % DBM in 0.1 M NH_4_HCO_3_ with GSH/GSSG) was significantly lower (~10–14 %) (Table 2). Göransson et al. have previously studied folding of cycloviolacin O2 and achieved ~40 % of native peptide (Leta Aboye et al. 2008), which is higher than what has been observed in this study using the DMSO/detergent buffer (11.4 %). However, their folding conditions were slightly different, i.e., (1) use of Brij 35 instead of DBM, (2) use of 2 mM cystamine in addition to GSH as redox component, (3) use of Tris instead of NH_4_HCO_3_-buffer, (4) folding temperature of 4 °C as well as 20 °C and (5) folding was allowed to proceed for 48 h with addition of fresh redox components after 24 h. To further analyse the influence of these differences folding of the control cyclotide cycloviolacin O2 in each folding condition was performed and in agreement with Göransson et al. we obtained the highest folding yield at 4 °C using the Tris-buffered 35 % DMSO/6 % Brij 35 after 48 h of folding (Table 2). In particular, the longer incubation time and lower temperature appeared to enhance the yield of natively folded cycloviolacin O2. Figure 7 shows the HPLC chromatograms of the highest yielding folding conditions for each cyclotide.

**Table 2:** Overview of yields and folding kinetics of vigno cyclotides

Cyclotide^a^	Buffer^b^	Yield^c^ (%)	Folding kinetics^d^		
			*k*_n_ (min^−1^)	*t*_1/2_ (min)	*R* ^2^
Vigno 1	25 % isopropanol (aqueous)	28.9	0.007	95.6	0.99
	75 % isopropanol (aqueous)	89.8	0.012	55.7	>0.99
	35 % DMSO/5 % dodecyl-β-maltoside (DBM)	62.7	0.010	69.6	0.93
Vigno 2	25 % isopropanol (aq.)	52.2	0.013	54.1	0.97
	75 % isopropanol (aq.)	80.4	0.027	25.6	0.99
	35 % DMSO/5 % DBM	30.5	0.015	46.1	0.89
Kalata B1	25 % isopropanol (aq.)	88.6	0.021	32.3	0.96
	75 % isopropanol (aq.)	87.7	0.018	37.7	0.99
	35 % DMSO/5 % DBM	87.0	0.009	75.2	>0.99
Vigno 10	25 % isopropanol (aq.)	1.3	–^e^	–	–
	75 % isopropanol (aq.)	11.4	–	–	–
	35 % DMSO/5 % DBM	1.0	–	–	–
Cycloviolacin O2	25 % isopropanol (aq.)	6.3	–	–	–
	75 % isopropanol (aq.)	13.5	–	–	–
	35 % DMSO/5 % DBM	11.4	–	–	–
	35 % DMSO/5 % DBM^f^	13.0	0.028	24.6	–
	35 % DMSO/6 % Brij35 (Tris, 4 °C)^f^	15.2/28.7 ^g^	0.008	92.4	>0.99
	35 % DMSO/6 % Brij35 (Tris, 20 °C)^f^	9.2/20.6 ^g^	0.032	21.7	0.99

^a^All folding experiments were carried out with peptide concentrations between 2.5 and 10 μM

^b^All buffers were prepared in 0.1 M NH_4_HCO_3_ (pH 8.2) with GSH/GSSG (2/0.1 mM) except where indicated otherwise

^c^Final yield after 24 h incubation at 20 °C; determined by automatic peak integration with a peak threshold set at 0.07 **×** signal (mAU) × RT (min) using Chromeleon software 6.8

^d^Rate constant of native folded peptide (*k*_n_) and folding half-time of observed folding yields determined by single exponential fit using GraphPad Prism 5, calculated over 24 h

^e^Not determined

^f^Buffer containing GSH/cystamine (2/2 mM)

^g^Yield after 48 h incubation, with addition of fresh GSH/cystamine (2/2 mM) after 24 h

Comparison of the sequences and structures provides insights into the differences observed in the folding efficiency in terms of yield and kinetics. Vigno 1 and vigno 2 have high sequence similarity to kalata B1, and all three peptides have high folding yields in 75 % isopropanol buffer (Table 2). However, there are differences in folding using the 25 % isopropanol and 35 % DMSO/detergent buffers (Table 2). The three Möbius peptides differ in loop 6, i.e., VRNGLPL in vigno 1, VRDGSSPL in vigno 2 and TRNGLPV in kalata B1 (Table 2; Supplementary Fig. S8). The two adjacent serine residues and an aspartic acid in vigno 2 make this loop more hydrophilic and confers the peptide an overall single negative net charge. This may explain higher folding yields of vigno 2 for the 25 % isopropanol buffer and lower yields for the more hydrophobic 75 % isopropanol buffer as compared to vigno 1, a neutral cyclotide. The more hydrophilic nature of loop 6 of vigno 2 (Supplementary Fig. S8) (Aboye et al. 2011) may also contribute to the different folding yields in the DMSO/detergent buffer, which vary between 62.7 % for vigno 1, 30.5 % for vigno 2 and 87 % for the control peptide kalata B1. Furthermore, loop 2 of vigno 1 and 2 are very similar; they both have a slightly more hydrophobic nature compared to kalata B1 (Supplementary Fig. S8), which probably also contributes to the folding differences and the overall later elution on RP-HPLC (Fig. 7f).

**Fig. 8:**
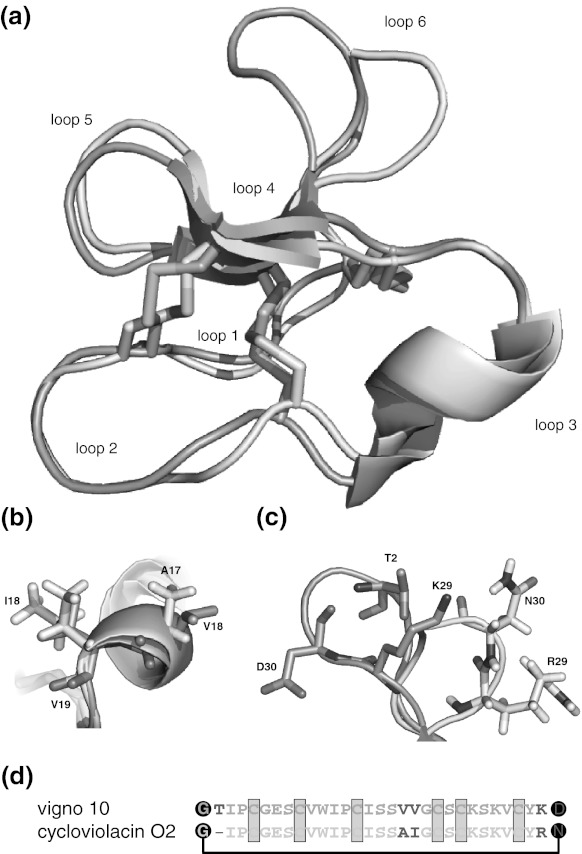
Structural alignment of vigno 10 and cycloviolacin O2. The structures of cycloviolacin O2 (*cyan* PDB code: 2GJ0) and the homology model of vigno 10 (*green*) were aligned using PyMOL (root-mean-square deviation = 1.324 Å) and are shown in *cartoon* representation (**a**). The cyclotide loops and disulfide bonds (*yellow*) are indicated. Differences in the side-chain orientation of the distinct residues of both cyclotides of loop 3 (**b**) and loop 6 (**c**) are indicated in *stick* representation and amino acids are labelled in one-letter code, numbered according to their position in the cyclotide sequence (starting from G1, see **d**). Images have been prepared using PyMOL. (**d**) Sequence alignment of vigno 10 and cycloviolacin O2 with the three conserved disulfide bridges (shown in *yellow*) and the cyclized backbone (*black dots* and *connecting line*). Residues differing between those two cyclotides have been highlighted in *red* (color figure online)

Analysis of the folding of the bracelet cyclotide vigno 10 highlights the complexity of the oxidative folding of this cyclotide sub-family. The folding yields of vigno 10 and cycloviolacin O2 in the 75 % isopropanol buffer were comparable (13.5 vs. 11.4 %, respectively), but considerably lower than that observed for the Möbius cyclotides. Interestingly, the use of the DMSO/detergent buffer did not increase the yield of correctly folded vigno 10, but in fact resulted in negligible amounts of the native fold. By contrast, this buffer resulted in approximately 11 % of the correctly folded cycloviolacin O2. Comparison of the sequences reveals that only loops 3 and 6 differ between vigno 10 and cycloviolacin O2. Loop 3 differs by two residues and loop 6 of vigno 10 is a hybrid between the loop 6 sequences of cycloviolacin O2 and kalata B8. Loop 3 forms a helical structure in bracelet cyclotides, and has previously been suggested to be important in the folding of cycloviolacin O2 (Göransson et al. 2009; Leta Aboye et al. 2008). The introduction of two valine residues in loop 3 of vigno 10, which does not favour the formation of helices, may account for slight distortion in this region of vigno 10 (Fig. 8). A study involving the synthesis of hybrids of kalata B1 and cycloviolacin O1 indicated that bracelet loops 2 and 6 significantly influence the folding (Gunasekera et al. 2009). Given the loop 2 sequences of vigno 10 and cycloviolacin O2 are identical, the differences in loop 6 are also likely to be involved in the folding differences observed for these two peptides. In the model of vigno 10 the backbone and side chain atoms of residues T2, K29 and D30 have different orientations as compared to residues R29 and N30 of cycloviolacin O2 (Fig. 8). These structural changes could contribute to the decreased folding yield in the DMSO/detergent buffer, by destabilizing a late folding intermediate, which shifts the equilibrium towards the non-native conformation (Leta Aboye et al. 2008). Furthermore, loop 2 contains an isoleucine previously shown to detrimentally influence the oxidative folding and this residue may, in part, be responsible for the generally lower yields observed for vigno 10 and cycloviolacin O2 with respect to the Möbius cyclotides. Taken together, our folding studies of the novel vigno cyclotides compared to kalata B1 and cycloviolacin O2 confirms the significant difference of Möbius versus bracelet folding and the strong influence of the surface characteristics of cyclotides and solution conditions on their folding.

## Conclusion

This work has broadened the knowledge about the immense sequence diversity of plant cyclotides, a unique class of naturally occurring backbone-cyclized peptides built around a conserved cyclic cystine-knot. By characterizing 13 sequences from an Iranian violet species it has been confirmed that cyclotides are one of the most abundant peptide class within the plant kingdom. The characterization of cyclotides in mixtures using MALDI-TOF/TOF analytics may overcome laborious isolation and challenges in de novo peptide sequencing. The use of different proteases as well as the assembly and alignment of sequence fragments facilitates the discovery of novel cyclotide sequences. In addition, by performing oxidative refolding studies on representative cyclotides the knowledge of their in vitro oxidative folding behaviour was extended and this underlines the high dependency of folding yield to their inter-cysteine loop sequences and careful choice of the folding conditions. These studies have further implications taking into account that cyclotides have numerous bioactivities and hence display a scaffold that is extensively used for peptide-based drug design.

## Electronic supplementary material

Below is the link to the electronic supplementary material.

Supplementary material 1 (PDF 1067 kb)

## Abbreviations

CCK: Cyclic cystine-knot
RP-HPLC: Reversed-phase high performance liquid chromatography
MS: Mass spectrometry
MALDI-TOF: Matrix-assisted laser desorption ionization-time of flight
endo-GluC: Endoproteinase GluC
DBM: Dodecyl-β-maltoside
DMSO: Dimethyl sulfoxide
GSH: Reduced glutathione
GSSG: Oxidized glutathione
SPE: Solid-phase extraction
FA: Formic acid
TFA: Trifluoroacetic acid
