# Supplementary material for: Characterizing circular peptides in mixtures: sequence fragment assembly of cyclotides from a violet plant by MALDI-TOF/TOF mass spectrometry
Source: Amino Acids. 2012 Aug 14;44(2):581–95. doi: 10.1007/s00726-012-1376-x (PMC3549257; doi:10.1007/s00726-012-1376-x)
Supplement: Supplementary file 1 — Supplementary material 1 (PDF 1067 kb) [file 726_2012_1376_MOESM1_ESM.pdf]

Supplementary Material

Characterizing circular peptides in mixtures: sequence fragment assembly of cyclotides from a violet plant by MALDI-TOF/TOF mass spectrometry

Hossein Hashempour, Johannes Koehbach, Norelle L. Daly, Alireza Ghassempour and Christian W. Gruber

Content:

Supplementary Tables S1-S3

Supplementary Figures S1-S10

Supplementary Table S1. *De novo* sequencing of Möbius cyclotides.

Cyclotide (fraction)	enzyme	amino acid sequence	Observed fragments/ precursor mass for MS/MS (Da)	L/I determination by (C^c/ homology[d])
**vigno 1 (1)**	E[a]	-TCAGGTCNTPGCSCSWPVCVRNGLPLCGE-	3227.1	C; homology
	T[b]	-NGLPLCGETCAGGTCNTPGCSCSWPVCVR-	3227.3	
	E + T	-TCAGGTCNTPGCSCSWPVCVR-	2386.9	
**vigno 2 (1)**	E	-TCAGGTCNTPGCSCSWPVCVRDGSSPLCGE	3289.1	C
	T	-GSSPLCGETCAGGTCNTPGCSCSWPVCVRD	3289.4	
	E + T	-TCAGGTCNTPGCSCSWPVCVR-	2386.9	
**vigno 3 (4)**	E	-TCVGGTCNTPGCSCSWPVCTRNGLPLCGE-	3257.3	C; homology
	T	-NGLPLCGETCVGGTCNTPGCSCSWPVCTR-	3257.1	
	E + T	-TCVGGTCNTPGCSCSWPVCTR-	2416.9	
**vigno 4 (4)**	E	-TCVGGTCNTPACSCSWPVCTRNGLPLCGE-	3271.1	C; homology
	T	-NGLPLCGETCVGGTCNTPACSCSWPVCTR-	3271.1	
	E + T	-TCVGGTCNTPACSCSWPVCTR-	2430.9	
**varv A (2)**	E	-TCVGGTCNTPGCSCSWPVCTRNGLPVCGE-	3243.0	C; homology
	T	-NGLPVCGETCVGGTCNTPGCSCSWPVCTR-	3242.1	
**vigno 5 (3)**	E	-TCVGGTCNTPGCSCGWPVCVRNGLPLCGE-	3225.1	C; homology
	T	-NGLPLCGETCVGGTCNTPGCSCGWPVCVR-	3225.1	

sequence characterization was carried out using the above listed peptides and fragments of various digests using [a]endoproteinase GluC, [b]trypsin, and [c]chymotrypsin, which cleaves peptides at the C-terminal side of leucine, but not isoleucine; [d]homology based on sequences as published on CyBase (www.cybase.org.au)

Supplementary Table S2. *De novo* sequencing of bracelet cyclotides by sequence fragment assembly.

Cyclotide (fraction)	enzyme	amino acid sequence	precursor mass for MS/MS (Da)	MS/MS (Yes/No)	L/I determination by (C[c]/homology[d])
**vigno 6 (6)**	E[a]	-SCVWIPCISSAIGCSCKGSKVCYRNGIPCGE-	3562.4	Yes	homology
	T[b]	-VCYRNGIPCGESCVWIPCISSAIGCSCK-	3290.1	Yes	homology
		-NGIPCGESCVWIPCISSAIGCSCK-	2711.9	Yes	
	C[c]	-RNGIPCGESCVW-	1434.5	No	C; homology
		-IPCISSAIGCSCKGSKVCY-	2146.5	No	
		-SSAIGCSCKGSKVCY-(theoretical Leu-fragment)	*1666.6*	*not present[e]*	
		-GCSCKGSKVCY-(theoretical Leu-fragment)	*1308.4*	*n.p.[e]*	
	E + C	-RNGIPCGE-	902.3	No	C; homology
		-IPCISSAIGCSCKGSKVCY-	2146.5	No	
**vigno 7 (7)**	E	-SCVWIPCISSVVGCSCKNKVCYKNGTIPCGE-	3619.1	Yes	homology
	T	-VCYKNGTIPCGESCVWIPCISSVVGCSCK-	3377.1	No	homology
		-NGTIPCGESCVWIPCISSVVGCSCK-	2827.9	Yes	
	C	-KNGTIPCGESCVW-	1507.5	No	C; homology
		-IPCISSVVGCSCKNKVCY-	2131.7	No	
		-SSVVGCSCKNKVCY-(theoretical Leu-fragment)	*1649.6*	*n.p.*	
	E + C	-IPCISSVVGCSCKNKVCY-	2131.6	No	C; homology
		-KNGTIPCGE-	976.3	No	
**vigno 8 (5)**	E	-SCVWIPCITSAVGCSCKSKVCYRNGIPCGE-	3505.3	Yes	homology
	T	-VCYRNGIPCGESCVWIPCISSAIGCSCK-	3290.1	Yes	homology
		-NGIPCGESCVWIPCISSAIGCSCK-	2711.9	Yes	
	C	-RNGIPCGESCVW-	1434.6	No	C; homology
		-IPCITSAVGCSCKSKVCY-	2089.9	No	
		-TSAVGCSCKSKVCY-(theoretical Leu-fragment)	*1608.6*	*n.p.*	
	E + C	-RNGIPCGE-	902.3	No	C; homology
		-IPCITSAVGCSCKSKVCY-	2089.6	No	
**c O2 (6)**	E	-SCVWIPCISSAIGCSCKSKVCYRNGIPCGE-	3505.3	yes	homology
	T	-VCYRNGIPCGESCVWIPCISSAIGCSCK-	3290.1	Yes	homology
		-NGIPCGESCVWIPCISSAIGCSCK-	2711.9	Yes	

	C	-RNGIPCGESCVW-	1434.5	No	C; homology
		-IPCISSAIGCSCKSKVCY-	2089.7	No	
		-SSAIGCSCKSKVCY- *(theoretical Leu-fragment)*	*1608.6*	*n.p.*	
	E + C	-RNGIPCGE-	902.2	No	C; homology
		-IPCISSAIGCSCKSKVCY-	2089.5	No	
**vigno 9 (6)**	E	-SCVWIPCISSALGCSCKSKVCYRNGIPCGE-	3505.3	Yes	homology
	T	-VCYRNGIPCGESCVWIPCISSALGCSCK-	3290.1	Yes	homology
		-NGIPCGESCVWIPCISSALGCSCK-	2711.9	Yes	
	C	-IPCISSAL-	860.4	No	C; homology
		-RNGIPCGESCVW-	1434.5	No	
		-GCSCKSKVCY-	1248.4	No	
		-IPCISSAIGCSCKVCY- *(theoretical Ile-fragment)*	*2093.9*	*n.p.*	
	E + C	-IPCISSAL-	860.4	No	C; homology
		-RNGIPCGE-	902.2	No	
		-GCSCKSKVCY-	1248.3	No	
**c O9 (7)**	E	-SCVWIPCLTSAVGCSCKSKVCYRNGIPCGE-	3505.3	Yes	homology
	T	-VCYRNGIPCGESCVWIPCLTSAVGCSCK-	3377.1	No	homology
		-NGIPCGESCVWIPCLTSAVGCSCK-	2711.9	Yes	
	C	-RNGIPCGESCVW-	1434.5	No	C; homology
		-TSAVGCSCKSKVCY-	1609.6	No	
		-IPCITSAVGCSCKSKVCY- *(theoretical Ile-fragment)*	*2089.7*	*n.p.*	
	E + C	-RNGIPCGE-	902.3	No	C; homology
		-TSAVGCSCKSKVCY-	1609.6	No	
**vigno 10 (7)**	E	-SCVWIPCISSVVGCSCKSKVCYKDGTIPCGE-	3593.4	Yes	homology
	T	-DGTIPCGESCVWIPCISSVVGCSCKSKVCYK-	2828.0	Yes	homology
	C	-IPCISSVVGCSCKSKVCYKDGTIPCGESCVW-	3593.0	Yes	C; homology[f]
		-IPCISSVVGCSCKSKVCY-	2103.9	Yes	C; homology
		-KDGTIPCGESCVW-	1508.6	Yes	Homology
		-SSVVGCSCKSKVCY- *(theoretical Leu-fragment)*	*1623.6*	*n.p.*	

sequence characterization was carried out using the above listed peptides and fragments of various digests using [a]endoproteinase GluC, [b]trypsin, and [c]chymotrypsin, which cleaves peptides at the C-terminal side of leucine, but not isoleucine; [d]homology based on sequences as published on CyBase (www.cybase.org.au); [e]fragment in MS spectra not present; [f]homology based on sequences of vigno 6, 8 and 9, as well as cycloviolacin O2 and O9

Supplementary Table S3. Molecular weight of cyclotides before and after folding experiments.

cyclotide	condition[a]	Observed MW[b]	Disulfide species
	Native[c]	2860.9	3SS
	Reduced[c]	2867.3	0SS
**vigno 1**	25% *iso*-PrOH (aqueous)	2861.0	3SS
	75% *iso*-PrOH (aqueous)	2861.0	3SS
	35% DMSO/5% DBM	2861.0	3SS
	Native	2922.9	3SS
	Reduced	2929.3	0SS
**vigno 2**	25% *iso*-PrOH (aq.)	2923.0	3SS
	75% *iso*-PrOH (aq.)	2923.0	3SS
	35% DMSO/5% DBM	2922.9	3SS
	Native	2890.9	3SS
	Reduced	2897.2	0SS
**kalata B1**	25% *iso*-PrOH (aq.)	2891.1	3SS
	75% *iso*-PrOH (aq.)	2891.0	3SS
	35% DMSO/5% DBM	2891.0	3SS
	Native	3227.1	3SS
	Reduced	3233.2	0SS
**vigno 10**	25% *iso*-PrOH (aq.)	3227.2	3SS
	75% *iso*-PrOH (aq.)	3227.2	3SS
	35% DMSO/5% DBM	3227.2	3SS
	Native	3139.1	3SS
	Reduced	3145.1	0SS
	25% *iso*-PrOH (aq.)	3139.4	3SS
	75% *iso*-PrOH (aq.)	3139.4	3SS
**cycloviolacin O2**	35% DMSO/5% DBM	3139.3	3SS
	35% DMSO/5% DBM[d]	3139.1	3SS
	35% DMSO/6% Brij (Tris, 4°C)[d, e]	3138.9	3SS
	35% DMSO/6% Brij (Tris, 20°C)[d, e]	3138.9	3SS

[a]all buffers were prepared in 0.1 M NH_4HCO_3 (pH 8.2) with GSH/GSSG (2/0.1 mM) except where indicated otherwise; [b]monoisotopic masses after 24 h of folding are listed, indicating the presence of fully reduced (0SS) or oxidized peptides (3SS) ; [c]0.1% TFA buffer; [d]buffer containing GSH/cystamine (2/2 mM); [e]after 24 h of folding

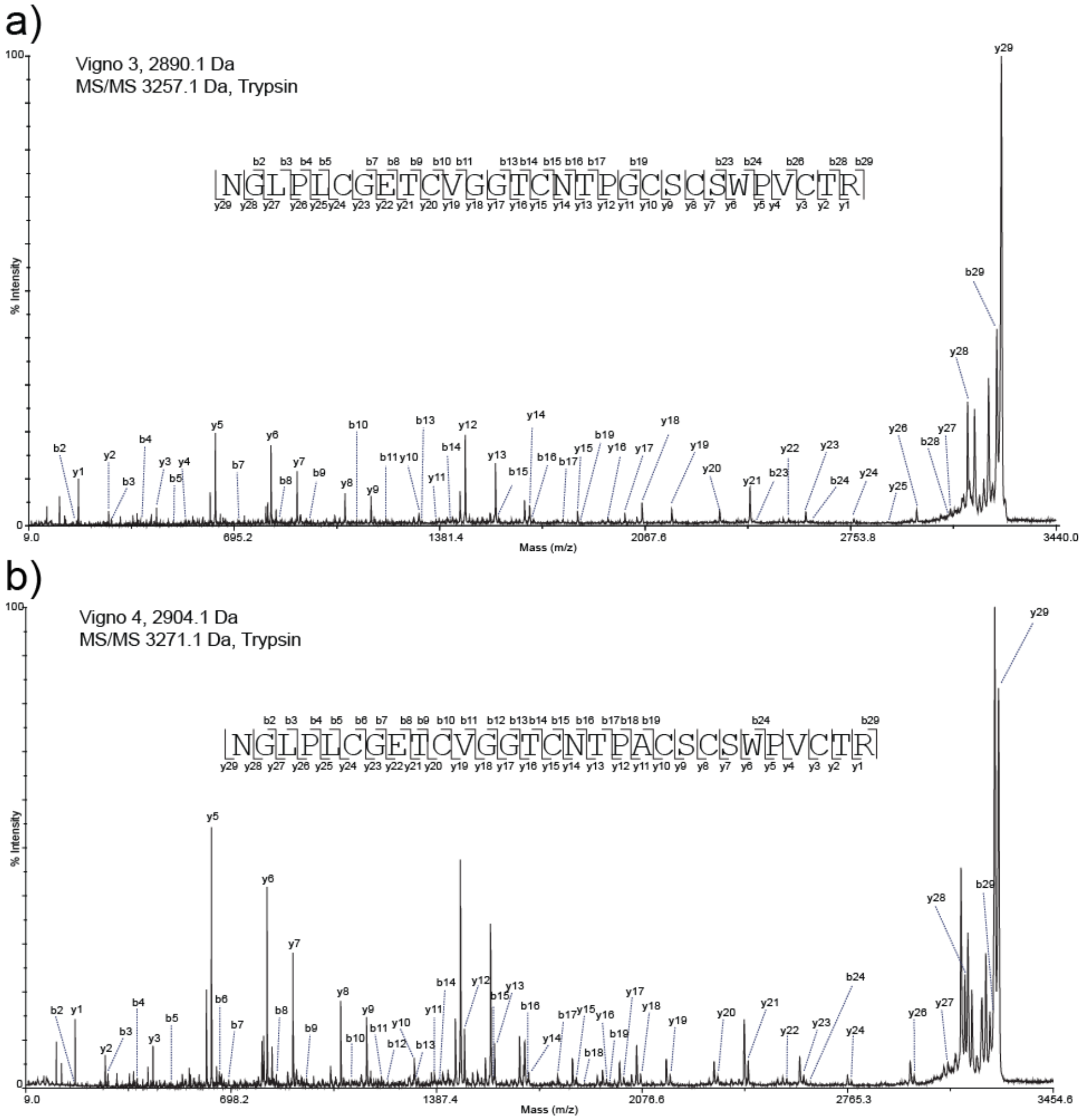

Supplementary Fig. S1. MALDI-TOF/TOF spectra for Möbius cyclotides vigno 3 (a) and vigno 4 (b). Observed N-terminal b- and C-terminal y-ions that allowed characterization of the displayed sequence are labelled.

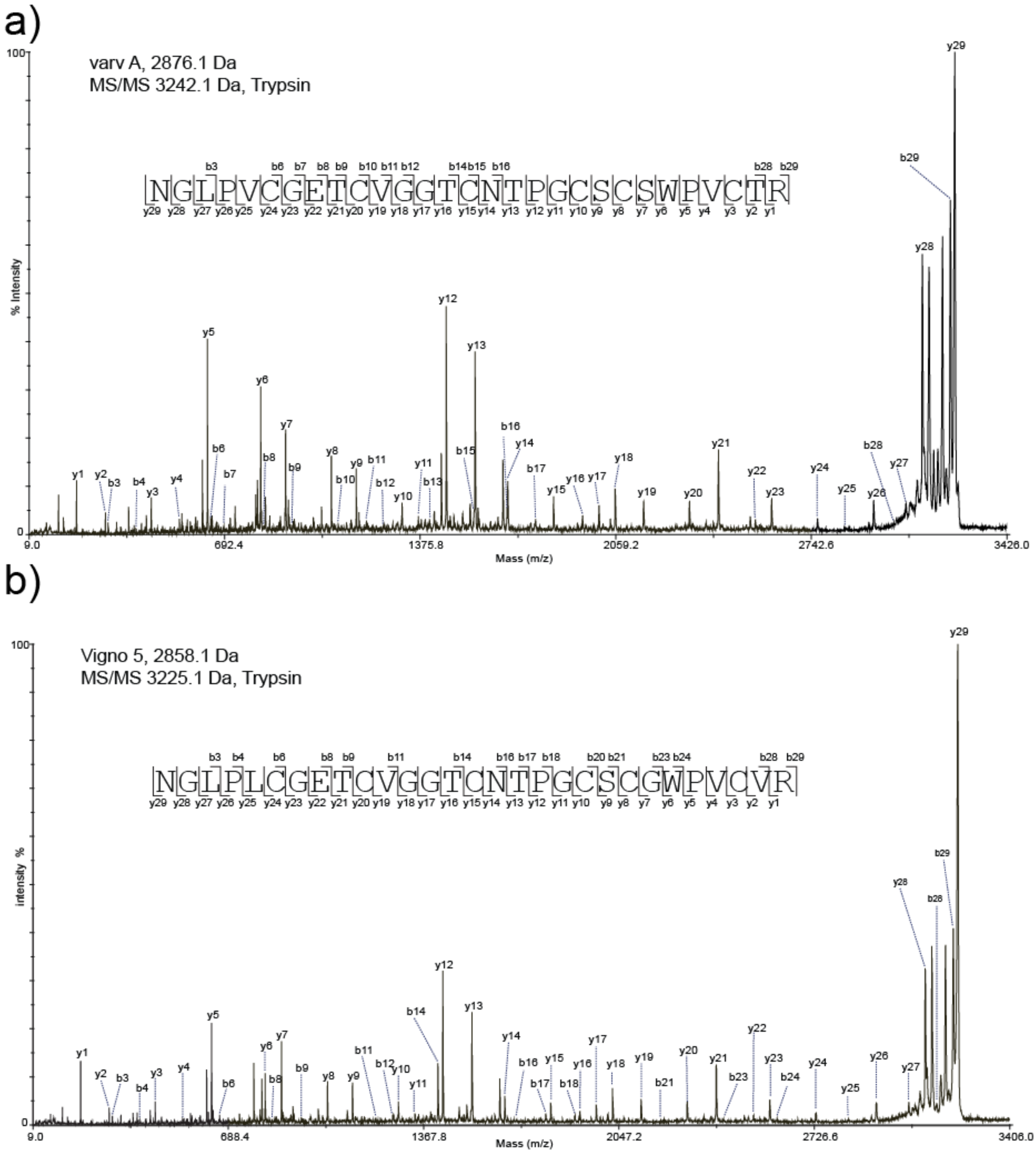

Supplementary Fig. S2. MALDI-TOF/TOF spectra for Möbius cyclotides varv A (a) and vigno 5 (b). Observed N-terminal b- and C-terminal y-ions that allowed characterization of the displayed sequence are labelled.

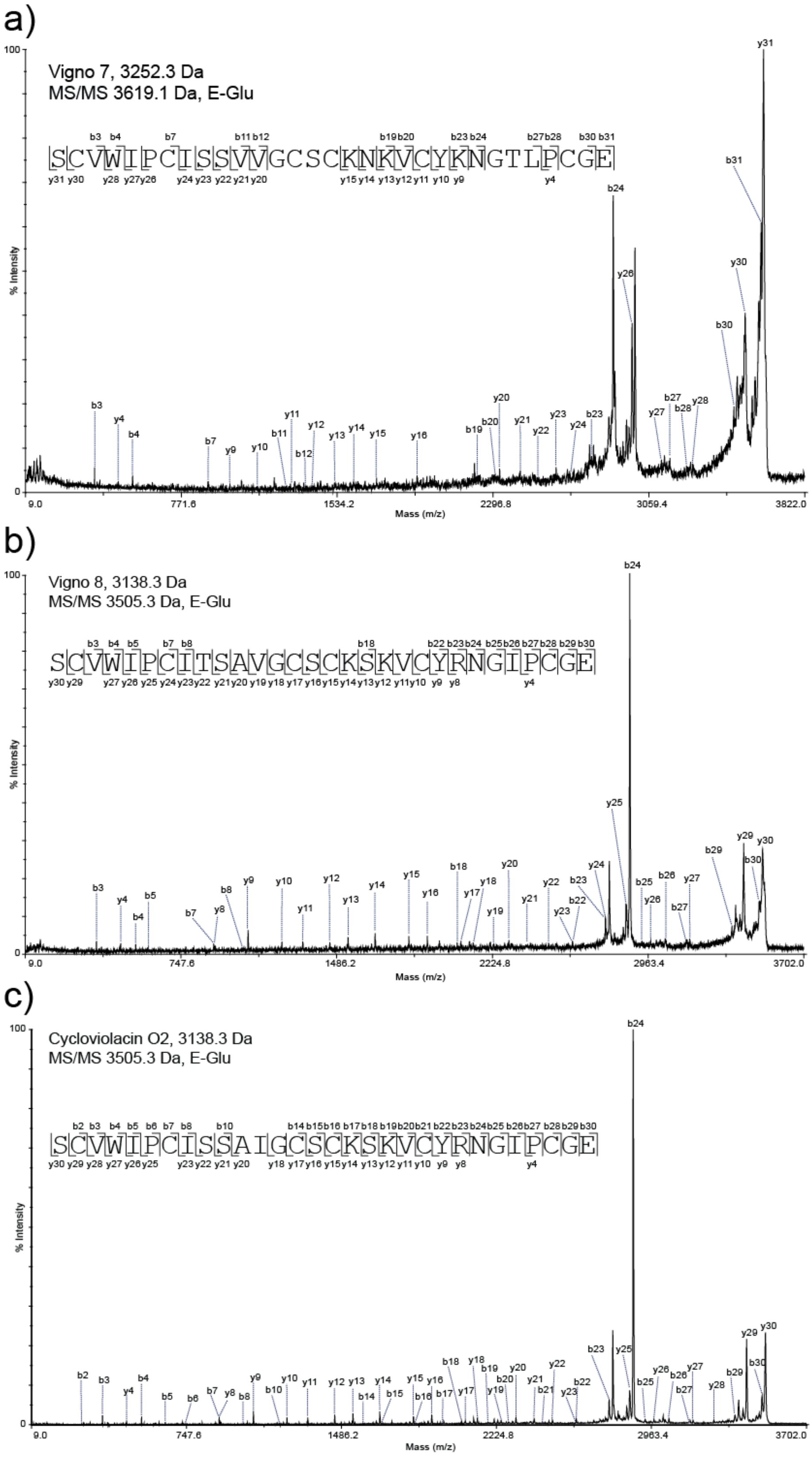

Supplementary Fig. S3. MALDI-TOF/TOF spectra for bracelet cyclotides vigno 7 (a), vigno 8 (b) and cycloviolacin O2 (c). Observed N-terminal b- and C-terminal y-ions that allowed characterization of the displayed sequence are labelled.

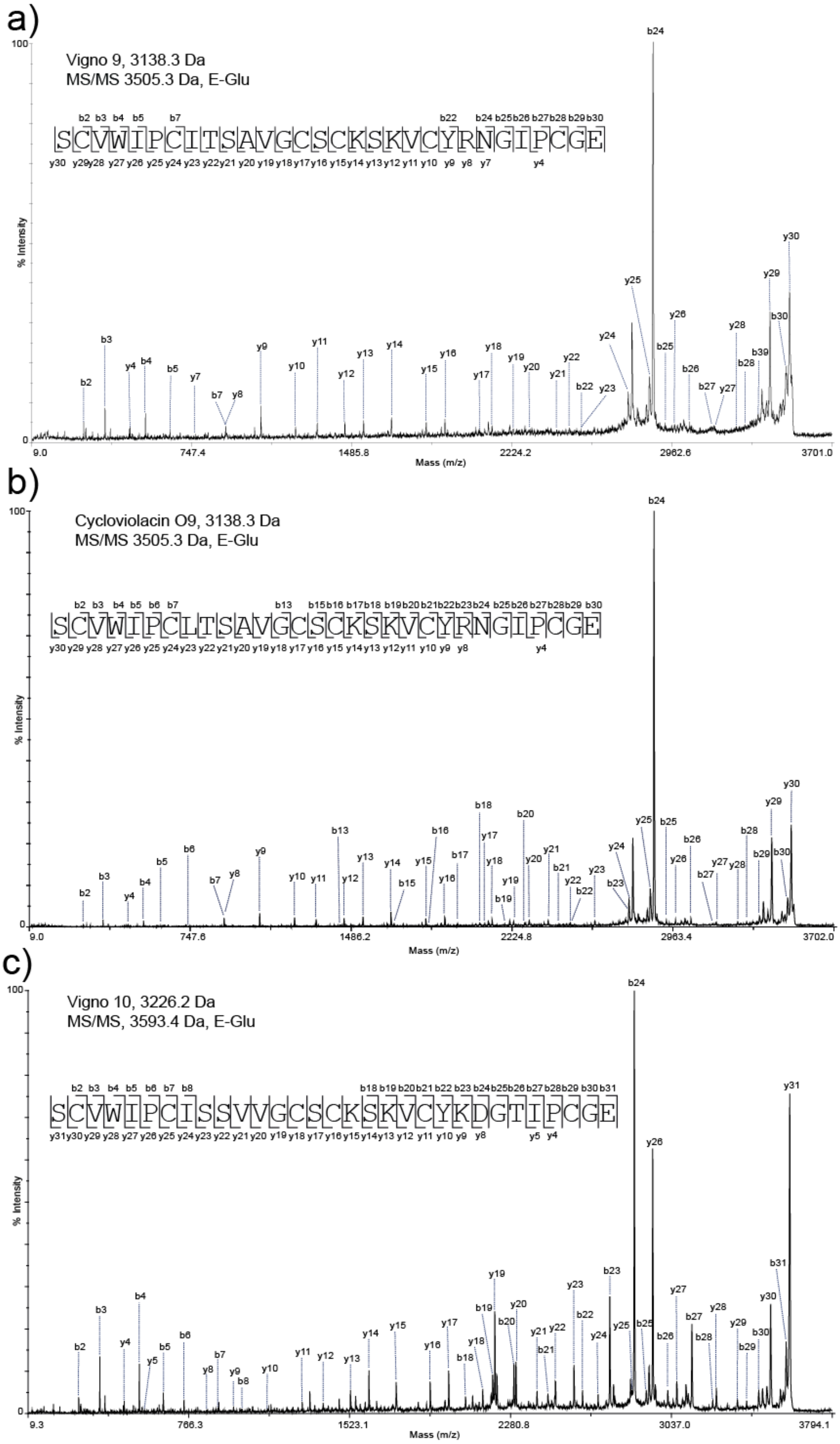

Supplementary Fig. S4. MALDI-TOF/TOF spectra for bracelet cyclotides vigno 9 (a), cycloviolacin O9 (b) and vigno 10 (c). Observed N-terminal b- and C-terminal y-ions that allowed characterization of the displayed sequence are labelled.

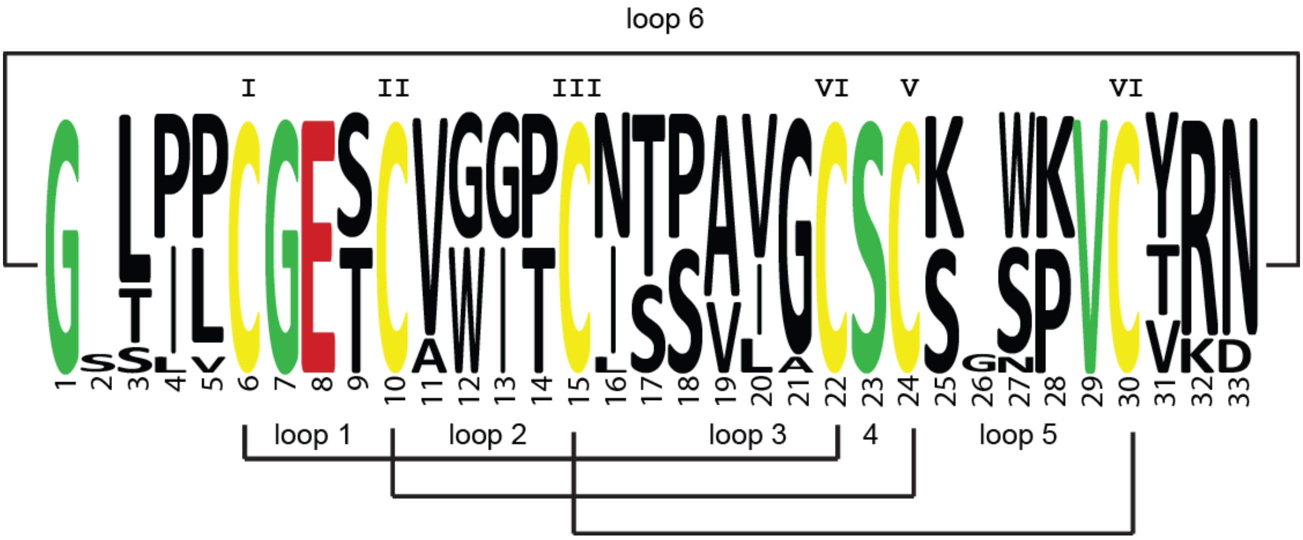

Supplementary Fig. S5. Sequence logo with relative frequency plot of novel *Viola ignobilis* cyclotides identified in this work. The highly conserved cysteine (yellow) and glutamic acid (red) residues are coloured and the cyclized backbone and the disulfide connectivity are shown. Furthermore the residues G1, G7, S23 and V29 are conserved within all identified cyclotides from *Viola ignobilis* and are coloured in green.

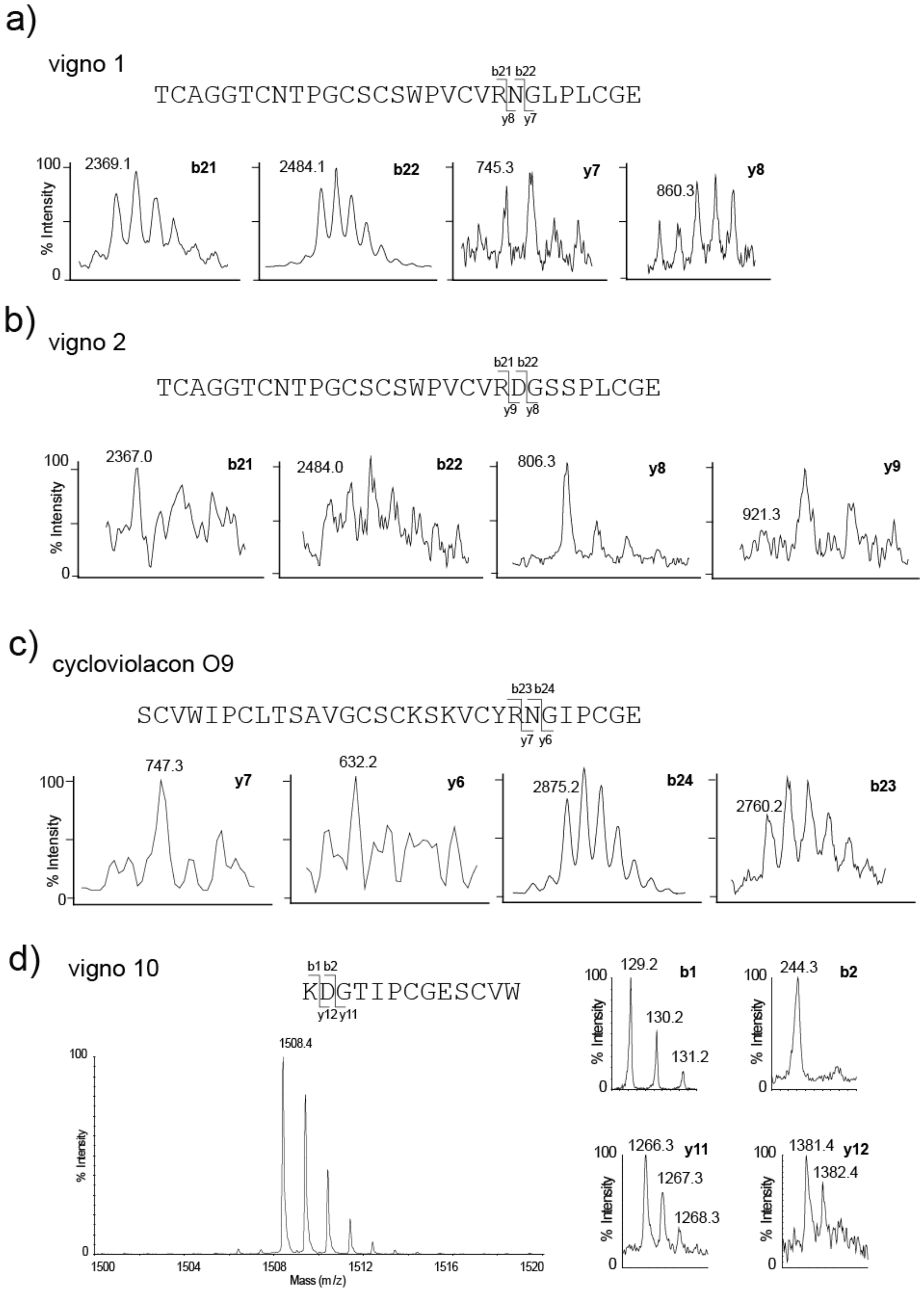

Supplementary Fig. S6. Isotopic distribution of Asn and Asp fragment ions are shown from different digests for vigno 1 (a), vigno 2 (b), cycloviolacin O9 (c) and vigno 10 (d). The molecular weight of specific N- or D-fragments ions and the isotope distribution of diagnostic fragment ions (as indicated) were analysed to confirm the presence of N, D or N/D mixtures in vigno cyclotides (see Poth et al. 2010).

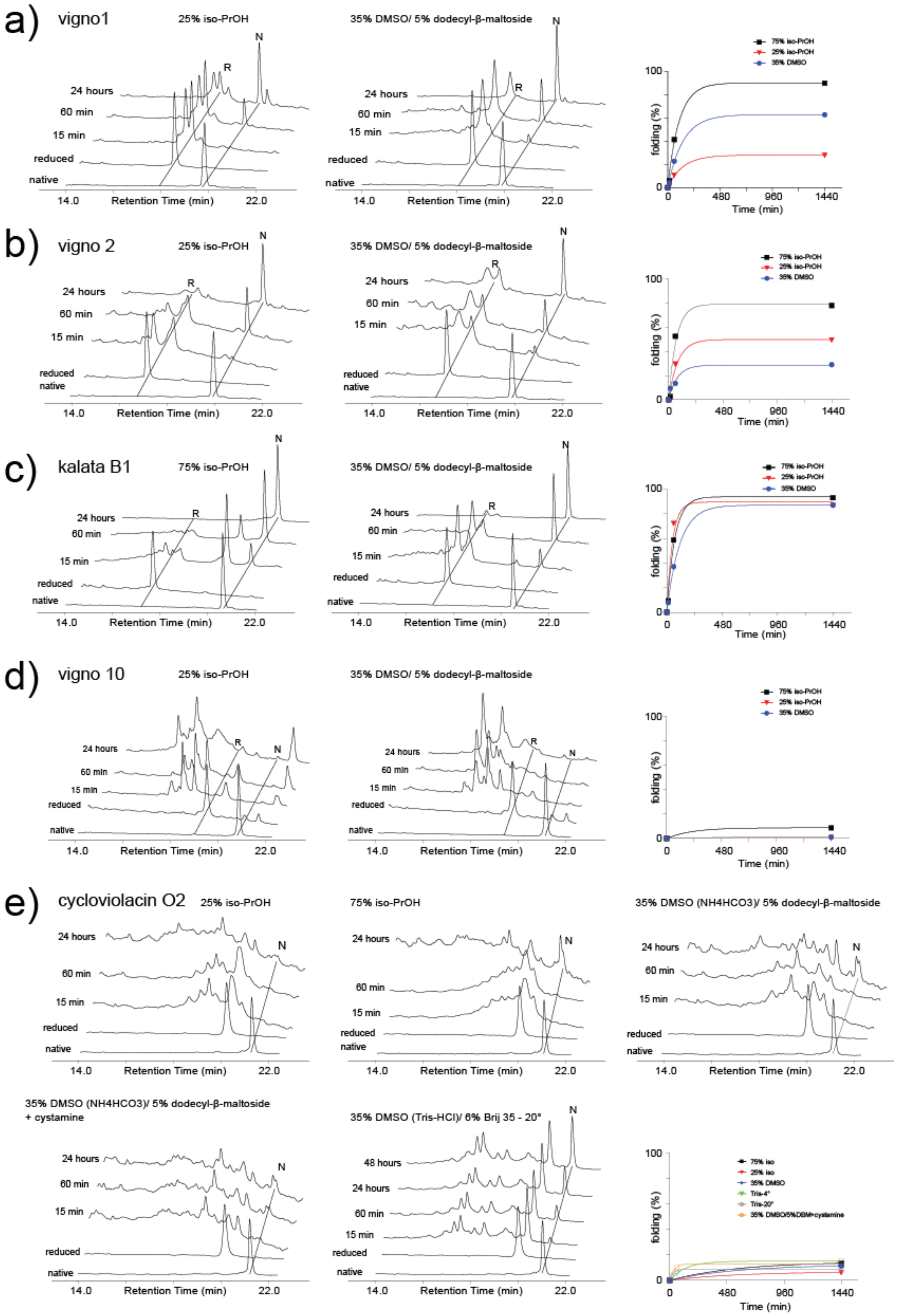

Supplementary Fig. S7. Refolding of novel vigno cyclotides. Vigno 1 (a), vigno 2 (b), vigno 10 (c), kalata B1 (d) and cycloviolacin O2 (e) are presented. RP-HPLC traces of native, reduced and refolded peptides are offset aligned. The folding of vigno peptides and kalata B1 (a-d) was performed in 0.1 M NH_4HCO_3 at 20°C and cycloviolacin O2 (e) folding was carried out in Tris-buffer at 20°C (see Materials and Methods for further details). Folding kinetic (right side panels) was calculated using GraphPad Prism 5 by plotting folding yields after 15, 60 and 1440 min determined by automatic peak integration using Chromeleon software.

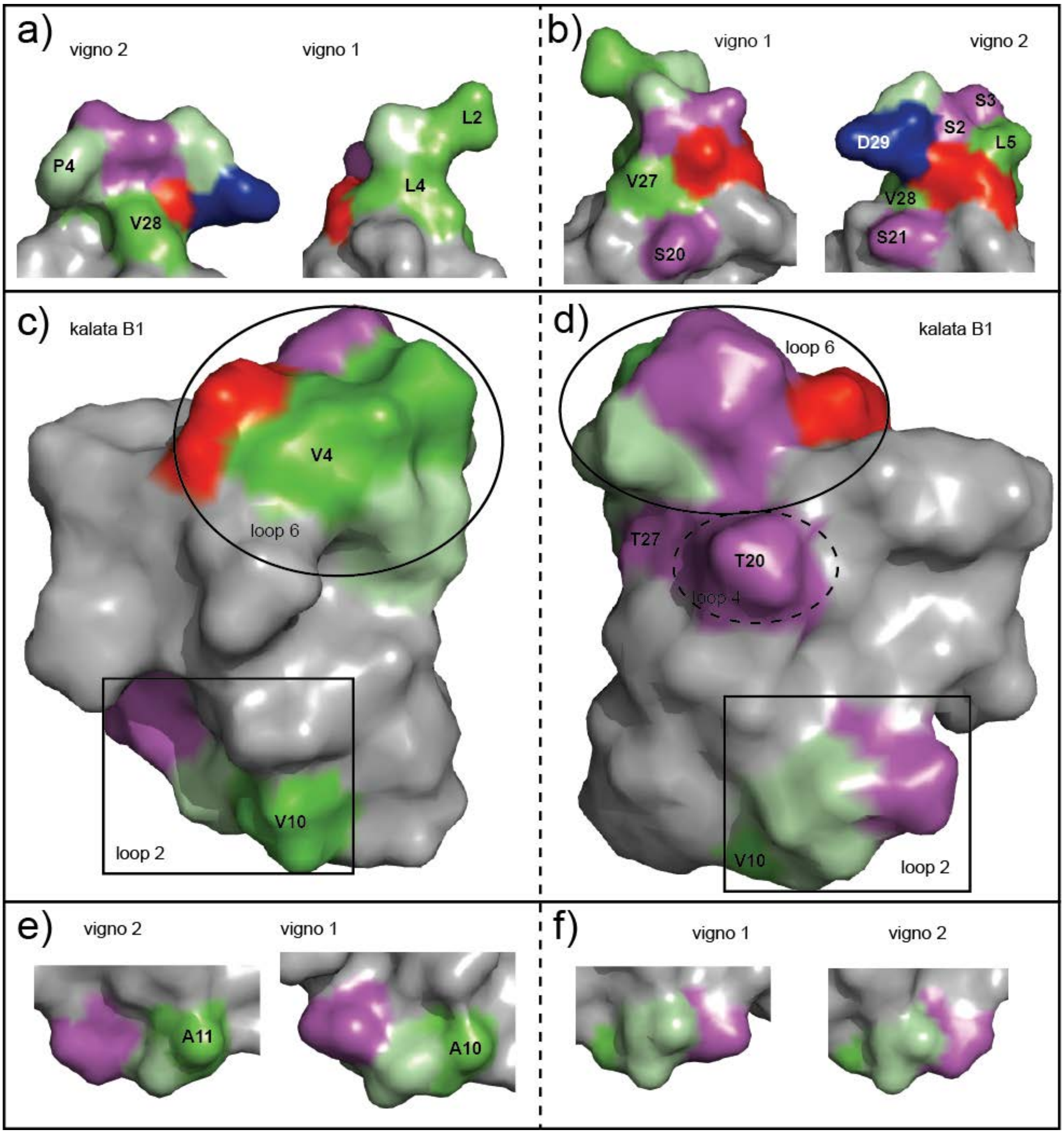

Supplementary Fig. S8. Surface representation of vigno 1, vigno 2 and kalata B1. Different surface properties of the homology models of vigno 1 and vigno 2 and the NMR structure of kalata B1 (PDB code: 1NB1) are illustrated. Vigno 1 and 2 differ in particular in loop 6 (a, b), loop 4 (b) and loop 2 (e, f). The surface characteristics of kalata B1 (c, d) with emphasis on loop 2 (solid rectangle), loop 4 (dashed circle) and loop 6 (solid circle) are shown for comparison. Hydrophobic residues are coloured in green, polar residues are coloured in magenta, basic residues are coloured in red and acidic residues are coloured in blue. Amino acids of interest are indicated in one-letter code and are numbered according to their position in the cyclotide sequence (starting from G1, see Table 2). Front (left; a, c, e) and back-side (right; b, d, f) images of the molecules are shown and have been separated by a dashed line. Images have been prepared using PyMOL.

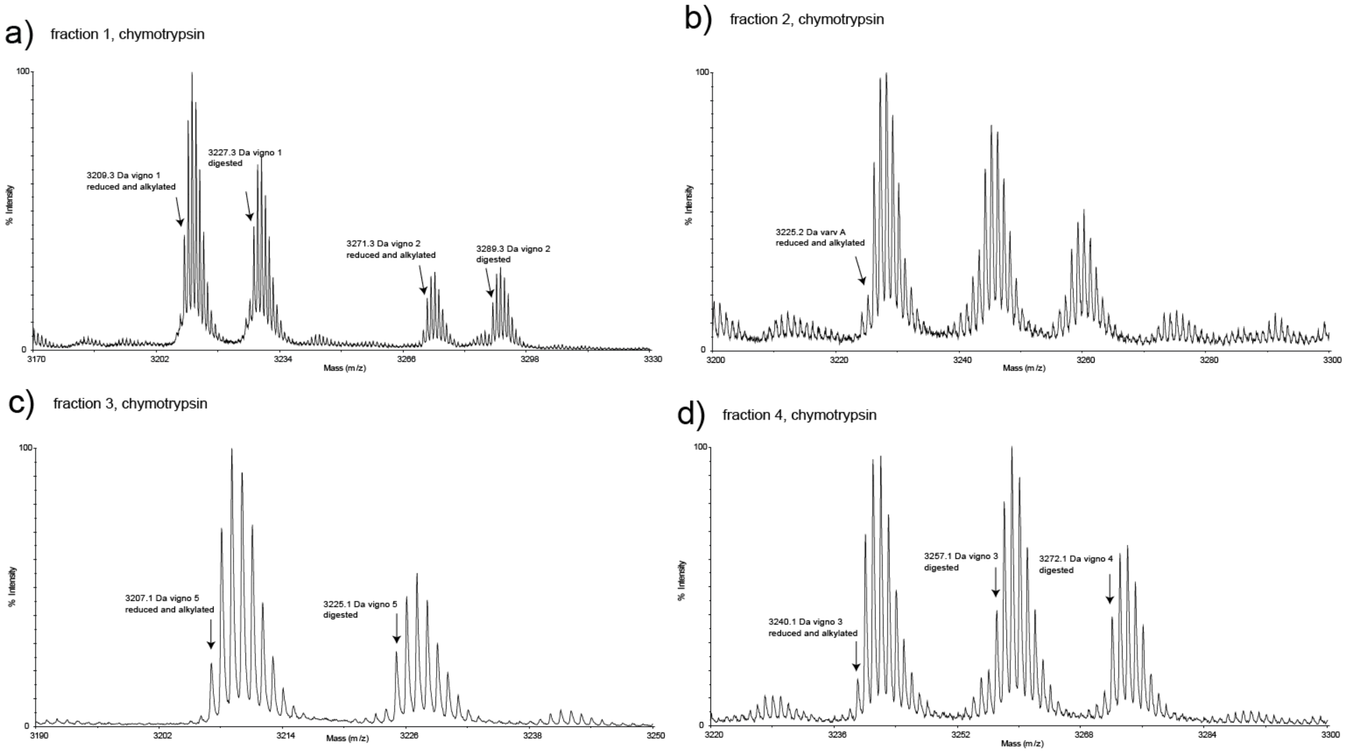

Supplementary Fig. S9. Chymotrypsin digest of Möbius cyclotides for the differentiation of isobaric Leu and Ile residues. Different cyclotide containing fractions 1 – 4 are shown (a-d) and the respective cyclotides are fragments are labelled.

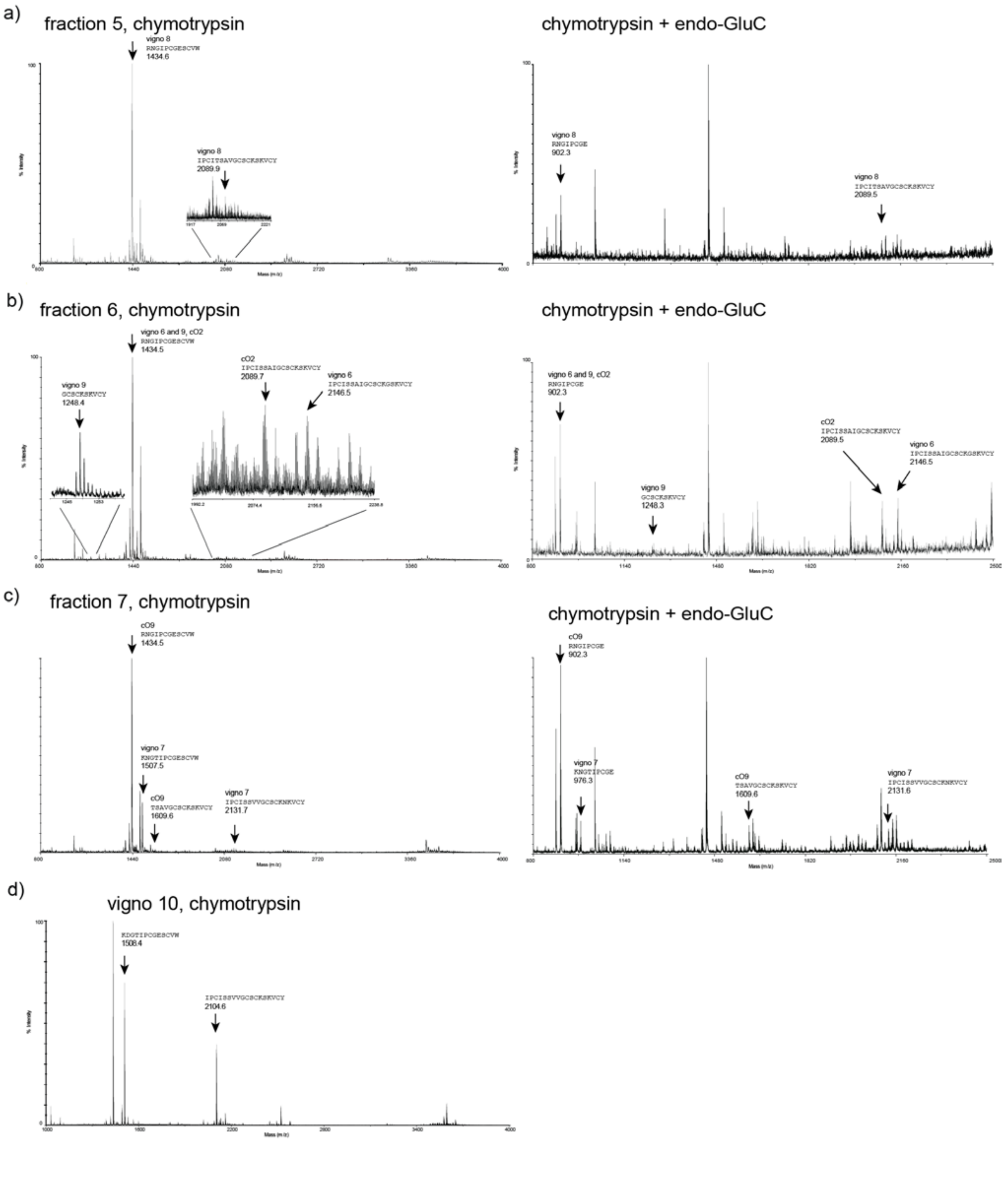

Supplementary Fig. S10. Chymotrypsin and chymotrypsin + endo-GluC digest of Möbius cyclotides for the differentiation of isobaric Leu and Ile residues. Cyclotide containing fractions 5 – 7 and a digest of pure vigno 10 are shown (a-d). Chymotrypsin digests (left side) and chymotrypsin and endoproteinase GluC double digests (right side) are labelled with the respective fragments that are expected based on their amino acid sequence.
